# Systematic Review on the Mechanisms of Action of Psilocybin in the Treatment of Depression

**DOI:** 10.1192/j.eurpsy.2023.898

**Published:** 2023-07-19

**Authors:** M. C. Q. Lin, H. Lee, V. W. L. Tsang, B. Chai, A. Howard, C. Uy, J. O. Elefante

**Affiliations:** 1 Faculty of Medicine; 2 Psychiatry; 3 Neurology, The University of British Columbia, Vancouver, Canada

## Abstract

**Introduction:**

Despite emerging evidence suggesting the efficacy of psilocybin in the treatment of mood disorders such as depression, the exact mechanisms by which psilocybin is able to elicit these antidepressant effects remains unknown.

**Objectives:**

As the use of psilocybin as a treatment modality for depression has garnered increasing interest, this study aims to summarize the existing evidence of the mechanism of action with which psilocybin alleviates depressive symptoms, focusing specifically on the neurobiological effects of psilocybin in human subjects.

**Methods:**

Four databases (Ovid MEDLINE, EMBASE, psychINFO, and Web of Science) were searched using a combination of MeSH terms and free text keywords in September 2021. The original search included both human and animal studies and must have included testing of the mechanism of action of psilocybin. Only antidepressant effects were considered, with no other mood disorders or psychiatric diagnoses included. Two independent researchers screened at every stage of the review, with a third researcher resolving any conflicts. Though a full systematic review outlining the current literature on the complete mechanisms of action of psilocybin on depression was conducted, this abstract will focus specifically on the nine papers that included human subjects, disregarding the five animal models. PROSPERO registration number: 282710.

**Results:**

After removing duplicates, the search identified 2193 papers and forty-nine were selected for full text review. Out of nine papers outlining the mechanisms of action of psilocybin use in human subjects, three papers investigated psilocybin’s effect on serotonin or glutamate receptor activity, two found an increase in synaptogenesis in regions such as the medial frontal cortex and hippocampus. Four found variation in blood flow to the amygdala, two found altered blood flow to the prefrontal cortex, and one found a reduction in delta power during sleep. Four papers found changes in functional connectivity or neurotransmission, most commonly in the hippocampus or prefrontal cortex.

**Conclusions:**

Overall, the exact mechanism of psilocybin’s potential antidepressant effect remains unclear. Multiple pathways may be involved, including alterations in serotonin and glutamate receptor activity, as well as shifts in amygdala activity, neurogenesis, and functional connectivity in various brain regions. The relative lack of studies, and the variety of neurobiological modalities and endpoints used challenged the consolidation of data into consensus findings. Further studies are needed to better characterize psilocybin’s mechanism of action and to better understand the clinical effects of the use of psilocybin in the treatment of depression.

**Disclosure of Interest:**

None Declared

